# Supplementary material for: A Study Evaluating Consumer Motivations, Perceptions, and Responses to Direct-to-Consumer Canine Genetic Test Results
Source: Animals (Basel). 2022 Nov 30;12(23):3360. doi: 10.3390/ani12233360 (PMC9741277; doi:10.3390/ani12233360)
Supplement: Supplementary file 1 [file animals-12-03360-s001.zip › animals-2031555-supplementary.pdf]

WP_UNLV Collaboration: Pet Owner Experience with Genetic Testing

---

Start of Block: Informed Consent

Informed Consent
Informed consent

UNLV Department of Anthropology

Title of Study: Social Science Research into Pet Owner Use of Direct-to-Consumer (DTC) Genetic Test Services

Investigator(s): Nikki Bennett & Dr. Peter Gray
For questions or concerns about the study, you may contact Nikki Bennett at **bennen2@unlv.nevada.edu** or Dr. Gray at peter.gray@unlv.edu. For questions regarding the rights of research subjects, any complaints or comments regarding the manner in which the study is being conducted, contact the UNLV Office of Research Integrity – Human Subjects at 702-895-2794, toll free at 888-581-2794 or via email at IRB@unlv.edu.

<u>Purpose of the Study</u>
You are invited to participate in a research study. The purpose of this study is to investigate consumer experience of using Mars Wisdom Panel's direct-to-consumer genetic test kits for pet dogs. These experiences are related to your motivations of using a dog genetic test, perception of your dog's test results, and response to your dog's genetic test results. Participants You are being asked to participate in the study because you fit these criteria: 18 years of age or older and have previously used a Wisdom Panel dog genetic test kit for a pet dog.

<u>Procedures</u>
If you volunteer to participate in this study, you will be asked to do the following: Complete an online survey anonymously.

<u>Benefits of Participation</u>
There will not be direct benefits to you as a participant in this study. However, we hope to learn more about people's use of animal genomic tests.

<u>Risks of Participation</u>
There are risks involved in all research studies. This study has minimal risks as this study does not collect information related to your identity. However, there are demographic questions asked and, while

unlikely, the combined responses have the potential to make an individual identifiable. If you are uncomfortable with answering any of these questions you may select “Prefer not to say.”

Cost/Compensation

There will be no financial cost to you to participate in this study. This study will take 5-10 minutes of your time. You will not be compensated for your time.

Confidentiality

All information gathered in this study will be kept as confidential as possible. No identifying information will be collected from you during the survey. No reference will be made in written or oral materials that could link you to this study. All records will be stored on a locked computer at UNLV for at least 3 years after completion of the study. After the storage time, the information gathered will either continue to be stored on a password protected computer or destroyed.

Voluntary Participation

Your participation in this study is voluntary. You may refuse to participate in this study or in any part of this study. You may withdraw at any time without prejudice to your relations with UNLV by closing your internet browser. You are encouraged to ask questions about this study at the beginning or any time during the research study.

Q1 **Participant Consent:**
I have read the above information and agree to participate in this study. I have been able to ask questions about the research study. I am at least 18 years of age. You may print this screen using your internet browser or you may download the form for your record here: Informed Consent Form

- ○ YES, I agree to participate in this study. (1)
- ○ NO, I do not agree to participate in this study. (2)

Skip To: End of Survey If Participant Consent: I have read the above information and agree to participate in this study. I... = NO, I do not agree to participate in this study.

End of Block: Informed Consent

Start of Block: Screening Question

Screening Question This survey will be asking about your experience with using Wisdom Panel’s direct-to-consumer genetic testing services for a dog.

Q2 Have you ever used a Wisdom Panel genetic test for a dog?

- ◯ YES, I have used genetic testing services for a dog. (1)
- ◯ NO, I have never used genetic testing services for a dog. (2)

Skip To: End of Survey If Have you ever used a Wisdom Panel genetic test for a dog? = NO, I have never used genetic testing services for a dog.

End of Block: Screening Question

Start of Block: Genetic Testing Services

Q3 Which Wisdom Panel test did you use for your dog?

- ◯ Essential (25+ health test results, ancestry/breed results, traits results) (1)
- ◯ Premium (Comprehensive health screening with 210 health test results, ancestry/breed results, traits results) (2)

Q4 How did you purchase your dog's genetic test?

- ◯ Purchased directly from Wisdom Panel webpage (1)
- ◯ Purchased from third party e-commerce website such as Amazon or Chewy.com (2)
- ◯ Was a gift to me from someone else (3)
- ◯ Other (please explain): (4) ____

Q5 How did you hear about Wisdom Panel?
Select all that apply.

- ☐ Recommended to me by someone who had previously used a pet genetic test (1)
- ☐ My veterinarian recommended I use a genetic test (2)
- ☐ I saw a video commercial such as on television (3)
- ☐ I saw an advertisement on a social media platform such as Facebook or Reddit (4)
- ☐ I received an advertisement through email (5)
- ☐ I received an advertisement in the mail (6)
- ☐ Someone I follow and/or am connected with on social media shared a post recommending (7)
- ☐ It was a gift to me (8)
- ☐ Other (please explain): (9) ____

End of Block: Genetic Testing Services

Start of Block: Pet Demographics

Q6&7 The following questions ask about your experience with using a Wisdom Panel genetic test for your dog. If you have tested more than one dog, we ask that you focus on your **most recent experience**.

If you would like to complete this survey for more than one dog, please feel free to retake the survey.

Q8 What is the name of the dog you had genetically tested?

____

Page Break

Q9 What is the sex of [dog’s name]?

- ◯ Intact/Unaltered Male (1)
- ◯ Neutered Male (2)
- ◯ Intact/Unaltered Female (3)
- ◯ Spayed Female (4)

Q10 How old is [dog’s name]?

For example, if your pet is 3 years and 1 month then enter 3 in the Years form field and 1 in the Months form field.

- ◯ Years (1) ____
- ◯ Months (2) ____

Q11 How long has [dog’s name] been a part of your family?

For example, if you have owned your pet for 6 months then enter 0 in the Years form field and 6 in the Months form field.

- ◯ Years (1) ____
- ◯ Months (2) ____

Q12 [dog’s name] is a (select one)

- ◯ Companion/family member (1)
- ◯ Part of a breeding program and not a pet (2)
- ◯ Both a companion animal and a part of a breeding program (3)
- ◯ Neither (please explain): (4) ____

Q13 How did you obtain [dog’s name]?

- ◯ Adopted from an animal rescue or shelter (1)
- ◯ Found as a stray (2)
- ◯ Purchased from a pet store (3)
- ◯ Purchased from an online ad such as Craigslist or Facebook marketplace (5)
- ◯ Purchased from a breeder (4)
- ◯ Was a gift to me from someone else (6)
- ◯ Adopted from someone I knew who could no longer keep them (7)
- ◯ From a litter of puppies my own dog had (8)
- ◯ Other (please explain): (9) ____

Q14 What breed is [dog’s name]?

You may select up to 5 breeds listed here. If the breed of your dog is not listed, please select "Other Not Listed."

- ☐ Airedale Terrier (4)
- ☐ Akita (177)
- ☐ Alaskan Malamute (178)
- ☐ American Bulldog (179)
- ☐ American Bully (Standard) (180)
- ☐ American Eskimo Dog (Miniature) (181)
- ☐ American Eskimo Dog (Standard) (182)
- ☐ Am. Staffordshire Terrier (183)
- ☐ American Pit Bull Terrier (184)
- ☐ Australian Cattle Dog (Heeler) (185)
- ☐ Australian Kelpie (186)
- ☐ Australian Shepherd (187)
- ☐ Australian Terrier (188)
- ☐ Barbet (189)
- ☐ Basenji (190)
- ☐ Basset Hound (191)
- ☐ Beagle (192)

- [ ] Beauceron (193)
- [ ] Bedlington Terrier (194)
- [ ] Belgian Malinois (195)
- [ ] Belgian Tervuren (196)
- [ ] Bernese Mountain Dog (197)
- [ ] Bichon Frise (198)
- [ ] Black and Tan Coonhound (199)
- [ ] Bloodhound (200)
- [ ] Bluetick Coonhound (201)
- [ ] Boerboel (202)
- [ ] Border Collie (203)
- [ ] Border Terrier (204)
- [ ] Boston Terrier (205)
- [ ] Bouvier des Flandres (206)
- [ ] Boxer (207)
- [ ] Boykin Spaniel (208)
- [ ] Bracco Italiano   (209)
- [ ] Briard (210)

- [ ] Brittany (211)
- [ ] Bull Terrier (Standard) (212)
- [ ] Bull Terrier (Miniature) (213)
- [ ] Bullmastiff (215)
- [ ] Cairn Terrier (216)
- [ ] Cane Corso (217)
- [ ] Cardigan Welsh Corgi (218)
- [ ] Catahoula Leopard Dog (219)
- [ ] Caucasian Shepherd (Ovcharka) (220)
- [ ] Cavalier King Charles Spaniel (221)
- [ ] Chesapeake Bay Retriever (222)
- [ ] Chihuahua (Long hair) (223)
- [ ] Chihuahua (Smooth) (224)
- [ ] Chinese Crested (225)
- [ ] Chinese Shar-Pei (226)
- [ ] Chinook (227)
- [ ] Chow Chow (228)
- [ ] Clumber Spaniel (229)

- ☐ Cocker Spaniel (American) (230)
- ☐ Cocker Spaniel (English) (231)
- ☐ Collie (Smooth) (232)
- ☐ Collie (Rough) (233)
- ☐ Coton De Tulear (234)
- ☐ Dachshund (smooth) (235)
- ☐ Dachshund (wire/long haired) (236)
- ☐ Dalmatian (237)
- ☐ Doberman Pinscher (238)
- ☐ Dogo Argentino (239)
- ☐ Dutch Shepherd (240)
- ☐ English Bulldog (351)
- ☐ English Setter (241)
- ☐ English Sheepdog (352)
- ☐ English Shepherd (242)
- ☐ English Springer Spaniel (243)
- ☐ English Toy Spaniel (244)
- ☐ English Toy Terrier (245)

- ☐ Eurasier (246)
- ☐ Field Spaniel (247)
- ☐ Finnish Lapphund (248)
- ☐ Finnish Spitz (249)
- ☐ Flat Coat Retriever (250)
- ☐ French Bulldog (251)
- ☐ German Pinscher (252)
- ☐ German Shepherd Dog (253)
- ☐ German Shorthaired Pointer (254)
- ☐ Giant Schnauzer (255)
- ☐ Glen of Imaal Terrier (256)
- ☐ Golden Retriever (257)
- ☐ Gordon Setter (258)
- ☐ Great Dane (259)
- ☐ Great Pyrenees (260)
- ☐ Greyhound (261)
- ☐ Harrier (262)
- ☐ Havanese (263)

☐ Irish Setter (264)

☐ Irish Terrier (265)

☐ Irish Wolfhound (266)

☐ Italian Greyhound (267)

☐ Japanese Chin (268)

☐ Japanese Spitz (269)

☐ Keeshond (270)

☐ Komondor (271)

☐ Kooikerhondje (272)

☐ Kuvasz (273)

☐ Labrador Retriever (274)

☐ Lagotto Romagnolo (275)

☐ Lancashire Heeler (276)

☐ Leonberger (277)

☐ Lhasa Apso (278)

☐ Maltese (279)

☐ Miniature American Shepherd (280)

☐ Miniature Pinscher (281)

- [ ] Miniature Schnauzer (282)
- [ ] Newfoundland (283)
- [ ] Norfolk Terrier (284)
- [ ] Norwich Terrier (285)
- [ ] Nova Scotia Duck Tolling Retriever (286)
- [ ] Old English Bulldog (287)
- [ ] Old English Sheepdog (288)
- [ ] Other Not Listed (348)
- [ ] Papillon (289)
- [ ] Parson Russell Terrier (290)
- [ ] Patterdale Terrier (Smooth or Broken) (291)
- [ ] Patterdale Terrier (Rough) (292)
- [ ] Pekingese (293)
- [ ] Pembroke Welsh Corgi (294)
- [ ] Pharaoh Hound (295)
- [ ] Plott (296)
- [ ] Pointer (English) (297)
- [ ] Pomeranian (298)

- ☐ Poodle (Miniature) (299)
- ☐ Poodle (Standard) (300)
- ☐ Poodle (Toy) (301)
- ☐ Portugese Water Dog (302)
- ☐ Presa Canario (303)
- ☐ Pug (304)
- ☐ Puli (305)
- ☐ Pumi (306)
- ☐ Rat Terrier (307)
- ☐ Redbone Coonhound (308)
- ☐ Rhodesian Ridgeback (309)
- ☐ Rottweiler (310)
- ☐ Russian Toy (311)
- ☐ Saluki (312)
- ☐ Samoyed (313)
- ☐ Schipperke (314)
- ☐ Scottish Deerhound (315)
- ☐ Scottish Terrier (316)

- Shetland Sheepdog (Sheltie) (317)
- Shiba Inu (318)
- Shih Tzu (319)
- Shiloh Shepherd (320)
- Siberian Husky (321)
- Silky Terrier (322)
- Smooth Fox Terrier (323)
- Soft Coated Wheaten Terrier (324)
- Spanish Water Dog (325)
- Spinone Italiano (326)
- St. Bernard (327)
- Standard Schnauzer (329)
- Swedish Vallhund   (330)
- Thai Ridgeback (331)
- Tibetan Mastiff (332)
- Tibetan Spaniel (333)
- Tibetan Terrier (334)
- Toy Fox Terrier (335)

- [ ] Treeing Walker Coonhound (336)
- [ ] Vizsla (337)
- [ ] Weimaraner (338)
- [ ] Welsh Springer Spaniel (339)
- [ ] West Highland White Terrier (340)
- [ ] Whippet (341)
- [ ] White Shepherd (342)
- [ ] Wire Fox Terrier (343)
- [ ] Wirehaired Pointing Griffon (344)
- [ ] Xoloitzcuintli (345)
- [ ] Yorkshire Terrier (346)

Q15 How long ago did you have [dog's name] genetically tested?

- ○ Within the last month (1)
- ○ Within the last year (2)
- ○ 1 to 3 years ago (3)

End of Block: Pet Demographics

Start of Block: Motivations

Q16 Please select the primary reason why you used a Wisdom Panel DNA test for [dog’s name].

- ○ To learn [dog’s name]'s breed results (1)
- ○ To learn [dog’s name]'s health results (2)
- ○ It was a gift to me from someone else (3)
- ○ Other (please explain): (4) ____

Q17 Please read the following statements and select the response that best applies to you.

The genomic information I received from Wisdom Panel about [dog's name] allowed me...

	Strongly Disagree (1)	Somewhat Disagree (2)	Neither Agree or Disagree (3)	Somewhat Agree (4)	Strongly Agree (5)
To satisfy my curiosity about [dog’s name]'s breed composition. (1)	○	○	○	○	○
To see if [dog’s name] was at risk for a specific disease. (2)	○	○	○	○	○
To learn about [dog’s name] without a veterinarian recommending the test. (3)	○	○	○	○	○
To learn about [dog’s name] at the recommendation of my veterinarian. (4)	○	○	○	○	○
To improve [dog’s name]'s health. (5)	○	○	○	○	○
To find out if [dog’s name] is at risk of having an adverse response to some common medications. (6)	○	○	○	○	○
To better plan for [dog’s name]'s future. (7)	○	○	○	○	○
To participate in something fun and entertaining. (8)	○	○	○	○	○

To satisfy my interest in genetics in general. (9)	○	○	○	○	○
To participate in research (10)	○	○	○	○	○
To learn more about [dog's name]'s background because I have limited information about [dog's name]. (11)	○	○	○	○	○
To make inferences about [dog's name]'s behavior (12)	○	○	○	○	○

End of Block: Motivations

Start of Block: Perception of Results

Q18 Please rate the following statements about your perceptions, attitudes, and beliefs towards the genetic test results for [dog's name].

	Strongly Disagree (1)	Somewhat Disagree (2)	Neither Agree nor Disagree (3)	Somewhat Agree (4)	Strongly Agree (5)
[dog's name]'s breed results were accurate. (1)	○	○	○	○	○
[dog's name]'s health results were accurate. (2)	○	○	○	○	○
[dog's name]'s trait results were accurate. (5)	○	○	○	○	○
Wisdom Panel genetic test results are trustworthy. (3)	○	○	○	○	○
I would recommend other dog owner's use the test I did for [dog's name]. (4)	○	○	○	○	○
The Wisdom Panel genetic test I used for [dog's name] has great value. (6)	○	○	○	○	○

End of Block: Perception of Results

Start of Block: Response to Results

Q20 Have you discussed [dog’s name]'s genetic test results with anyone?

○ Yes (1)

○ No (2)

End of Block: Response to Results

Start of Block: Discussed Results with Someone

Display This Question:

If Have you discussed ${q://QID8/ChoiceTextEntryValue}'s genetic test results with anyone? = Yes

Q21 With whom did you discuss [dog’s name]'s genetic test results? (Select all that apply)

▢ Family member(s) (12)

▢ Friend(s) (13)

▢ Co-workers / Colleagues (3)

▢ Veterinarian not employed by Wisdom Panel (14)

▢ Wisdom Panel customer service (15)

▢ Wisdom Panel veterinarian/genetic counsellor (5)

▢ A genetic specialist / counselor not employed by Wisdom Panel (7)

▢ Other animal-related professional (8)

▢ Contacts on social networking services (e.g., Facebook, Reddit) (9)

▢ Other (please explain): (10) ____

Display This Question:

If Have you discussed ${q://QID8/ChoiceTextEntryValue}'s genetic test results with anyone? = Yes

Q22 What aspects of [dog’s name]'s genetic test results did you discuss? (Select all that apply)

- ▢ Breed results (1)
- ▢ Health-related results (2)
- ▢ Carrier status of certain traits (3)
- ▢ Other (please explain): (4) ____

End of Block: Discussed Results with Someone

Start of Block: Discussed Results with No One

Display This Question:

If Have you discussed ${q://QID8/ChoiceTextEntryValue}'s genetic test results with anyone? = No

Q23 Why have you NOT discussed [dog’s name]'s genetic test result with anyone? (Select all that apply).

- ▢ I don't feel that [dog’s name]'s results are important enough to share. (1)
- ▢ I don't think anyone else is interested in [dog’s name]'s results. (2)
- ▢ I am concerned about how others would react to [dog’s name]'s results (3)
- ▢ I plan to discuss [dog’s name]'s results with a veterinarian or other animal-related professional but haven't gotten around to it. (4)
- ▢ I plan to discuss [dog’s name]'s results with friends and family but haven't gotten around to it. (5)
- ▢ Other (please explain): (6) ____

End of Block: Discussed Results with No One

Start of Block: Response to Results Cont.

Q24 After receiving [dog’s name]'s genetic test results, what did you do with the results? (Select all that apply)

- ▢ I altered the ways in which I care for [dog’s name] such as their diet. (6)
- ▢ I altered [dog’s name]'s training. (7)
- ▢ I altered [dog’s name]'s medical care. (8)
- ▢ I did nothing with the test results. (4)
- ▢ Other (please explain): (9) ____

Q25 If you had a question about [dog's name]'s genetic test results, whom would you consult? (Select all that apply)

- ▢ Spouse / Significant other (1)
- ▢ Other family member(s) (2)
- ▢ Friend(s) (3)
- ▢ A veterinary care provider (4)
- ▢ Wisdom Panel customer service (5)
- ▢ Wisdom Panel veterinarian / genetic counselor (6)
- ▢ Health and medical websites (7)
- ▢ The breeder and/or store I purchased [dog's name] from (8)
- ▢ The organization I adopted [dog's name] from (9)
- ▢ Groups on social media like Facebook or Reddit (10)
- ▢ [dog's name]'s groomer (11)
- ▢ [dog's name]'s trainer (12)
- ▢ I would not consult with anyone (13)
- ▢ Other (please explain): (14) ____

End of Block: Response to Results Cont.

Start of Block: Demographics

Q28 What is your sex?

- ○ Male (1)
- ○ Female (2)
- ○ Non-binary / third gender (3)
- ○ Prefer not to say (4)

Q29 What is your age range?

- ○ 18-25 (1)
- ○ 26-35 (2)
- ○ 36-45 (3)
- ○ 46-55 (4)
- ○ 56-65 (5)
- ○ Older than 65 (6)

Q30 What is your racial/ethnic background?

- ○ White (1)
- ○ Black or African American (2)
- ○ American Indian or Alaska Native (3)
- ○ Latino or Hispanic (8)
- ○ Asian (4)
- ○ Native Hawaiian or Pacific Islander (5)
- ○ Other (6)
- ○ Prefer not to say (7)

Q31 What is your annual household income?

- ○ Less than $30,000 (1)
- ○ $30,000 - $59,999 (2)
- ○ $60,000 - $89,999 (7)
- ○ $90,000 - $129,999 (10)
- ○ $130,000 - $149,999 (11)
- ○ $150,000 - $199,999 (13)
- ○ More than $200,000 (14)
- ○ Prefer not to say (12)

Q32 What is your highest level of education?

- ○ Less than high school (1)
- ○ High school graduate or GED equivalent (2)
- ○ Some college (3)
- ○ Associates degree (4)
- ○ Technical or Vocational Training (5)
- ○ Bachelor's Degree (6)
- ○ Master's Degree (7)
- ○ Doctorate or Professional Degree (8)
- ○ Prefer not to say (9)

End of Block: Demographics
